# Application of early warning signs to physiological contexts: a comparison of multivariate indices in patients on long-term hemodialysis

**DOI:** 10.3389/fnetp.2024.1299162

**Published:** 2024-03-26

**Authors:** Véronique Legault, Yi Pu, Els Weinans, Alan A. Cohen

**Affiliations:** ^1^ Research Center of the Centre Hospitalier Universitaire de Sherbrooke, Sherbrooke, QC, Canada; ^2^ PRIMUS Research Group, Department of Family Medicine, University of Sherbrooke, Sherbrooke, QC, Canada; ^3^ Copernicus Institute of Sustainable Development, Environmental Science, Faculty of Geosciences, Utrecht University, Utrecht, Netherlands

**Keywords:** critical transition, biomarkers, mortality, variance, autocorrelation, cross-correlation, network physiology

## Abstract

Early warnings signs (EWSs) can anticipate abrupt changes in system state, known as “critical transitions,” by detecting dynamic variations, including increases in variance, autocorrelation (AC), and cross-correlation. Numerous EWSs have been proposed; yet no consensus on which perform best exists. Here, we compared 15 multivariate EWSs in time series of 763 hemodialyzed patients, previously shown to present relevant critical transition dynamics. We calculated five EWSs based on AC, six on variance, one on cross-correlation, and three on AC and variance. We assessed their pairwise correlations, trends before death, and mortality predictive power, alone and in combination. Variance-based EWSs showed stronger correlations (r = 0.663 ± 0.222 vs. 0.170 ± 0.205 for AC-based indices) and a steeper increase before death. Two variance-based EWSs yielded HR95 > 9 (HR95 standing for a scale-invariant metric of hazard ratio), but combining them did not improve the area under the receiver-operating curve (AUC) much compared to using them alone (AUC = 0.798 vs. 0.796 and 0.791). Nevertheless, the AUC reached 0.825 when combining 13 indices. While some indicators did not perform overly well alone, their addition to the best performing EWSs increased the predictive power, suggesting that indices combination captures a broader range of dynamic changes occurring within the system. It is unclear whether this added benefit reflects measurement error of a unified phenomenon or heterogeneity in the nature of signals preceding critical transitions. Finally, the modest predictive performance and weak correlations among some indices call into question their validity, at least in this context.

## 1 Introduction

Critical transitions are defined as abrupt and irreversible changes of the state or integrity of a complex system, leading to either disruption of the system or emergence of an alternative state (or regime shift). Although sudden in appearance, critical transitions are preceded by a characteristic phase, named critical slowing down, during which several dynamic changes in the system can be detected. The most notable features of the critical slowing down are a slower rate of recovery from perturbations (Wissel, 1984), as well as a persistent increase in variance, autocorrelation (AC), and cross-correlation among different elements of the system (also called spatial correlation in ecology; Scheffer et al., 2009). Early warning signals (EWSs) have been developed to capture these dynamic changes, usually reflecting B-tipping scenarios indicating bifurcation of the system (Ashwin, 1999; Ashwin et al., 2012; Perryman and Wieczorek, 2014), and have been widely used to study critical transitions in diverse systems, including ecosystems (Wouters et al., 2015; Pedersen et al., 2017; Xu et al., 2023), financial markets (Diks et al., 2019; Tu et al., 2020; Ismail et al., 2022), and climate systems (Dakos et al., 2008; Dylewsky et al., 2023). Application to clinical contexts is also gaining a growing interest, with applications in tumor detection (Xu et al., 2022; Zhong et al., 2022; Huang et al., 2023), detection of emerging infectious diseases (Chen P. et al., 2019; Brett et al., 2020; Li et al., 2022; Proverbio et al., 2022), mental disorders (van de Leemput et al., 2014; Bayani et al., 2017; Bos et al., 2022), sepsis (Tambuyzer et al., 2014; Almeida and Nabney, 2016; Ghalati et al., 2019), environmental health (Wang et al., 2018), alcohol use disorders (Foo et al., 2017), epileptic seizures (Maturana et al., 2020; Karasmanoglou et al., 2023), intestinal health (Lahti et al., 2014), and chronic diseases (Venegas et al., 2005; Li et al., 2014; Liu et al., 2021; Cohen et al., 2022). Moreover, Nazarimehr et al. (2020) review on EWSs offers useful perspective on their applications and limitations, including in biological systems.

From a network physiology perspective (Ivanov, 2021; Schöll, 2022), multivariate EWSs are of interest because they may reflect the integration of multiple physiological signals related to stability of the organism and/or shifts between discrete physiological states (Nakazato et al., 2020; Liu et al., 2021; Cohen et al., 2022). While critical transitions may reflect system collapse, they might also reflect controlled transitions between states such as breeding and non-breeding or awake and asleep (Yang et al., 2016; Ivanov and Bartsch, 2022); understanding the dynamics of critical transitions in physiology and how to predict them thus has relevance for understanding which physiological transitions are the results of programming along a clear pathway, and which are emergent phenomena from the system dynamics. It also, obviously, has relevance for understanding the dynamics of physiological collapse and risk of death (Cohen et al., 2022). In particular, previous findings show that at least some physiological systems show synchronization of variability across system compartments prior to critical transitions; multivariate EWSs thus may shed light on synchronicity in network physiology (Cohen et al., 2022; Garcia-Retortillo and Ivanov, 2022; Hasselman et al., 2023).

Some discrepancy among the findings on EWSs still exists (Milanowski and Suffczynski, 2016; Wilkat et al., 2019; O’Brien and Clements, 2021; Schreuder et al., 2022). For instance, earlier applications in physiology, mostly performed using univariate measures, showed variance and AC measures of heart rate variability as good predictors of mortality (Ho et al., 1997), but less conclusive results for an AC measure of EEG signals to anticipate the onset of epileptic seizures (Martinerie et al., 1998). Furthermore, application to real-world data raises several new challenges not considered in theorical models (Brett et al., 2018; Southall et al., 2021; Dablander et al., 2022), such as variation in temporal resolution (Clements and Ozgul, 2018), as well as random (Hillebrand et al., 2020) and extrinsic noise (Qin and Tang, 2018). Although various EWSs have been proposed, with a growing interest for multivariate EWSs over the past years (Eason et al., 2016; Chen S. et al., 2019; Laitinen and Lahti, 2022), few studies opted for a comparative approach (but see Dakos et al., 2012a; Weinans et al., 2021). As the performance of EWSs might well be context-dependent (Boettiger and Hastings, 2012; Weinans et al., 2021), comparative approaches to specific contexts/datasets could be very informative.

Over the past few years, we have developed two different multivariate EWSs, both highly predictive of mortality in a retrospective cohort of patients with chronic kidney disease (CKD) on long-term hemodialysis (Liu et al., 2021; Cohen et al., 2022). The first one, named “MMD” for multivariate moving distance, is based on statistical distance, using the previous observation as the reference in the calculation to measure intra-individual change over time (Liu et al., 2021). High MMD is indicative of high variance but low autocorrelation. The second consists of the scores of the first axis of a principal component analysis (PCA) on all the coefficients of variation (CVs) of selected biomarkers, thus called “CVPC1” (Cohen et al., 2022). It is thus a summary of the overall variability of the system. While some EWSs have been compared in simulation studies (Dakos et al., 2012a; Weinans et al., 2021), comparisons with these newly developed EWSs have yet to be performed, and none have been compared in empirical data.

**FIGURE 1:**
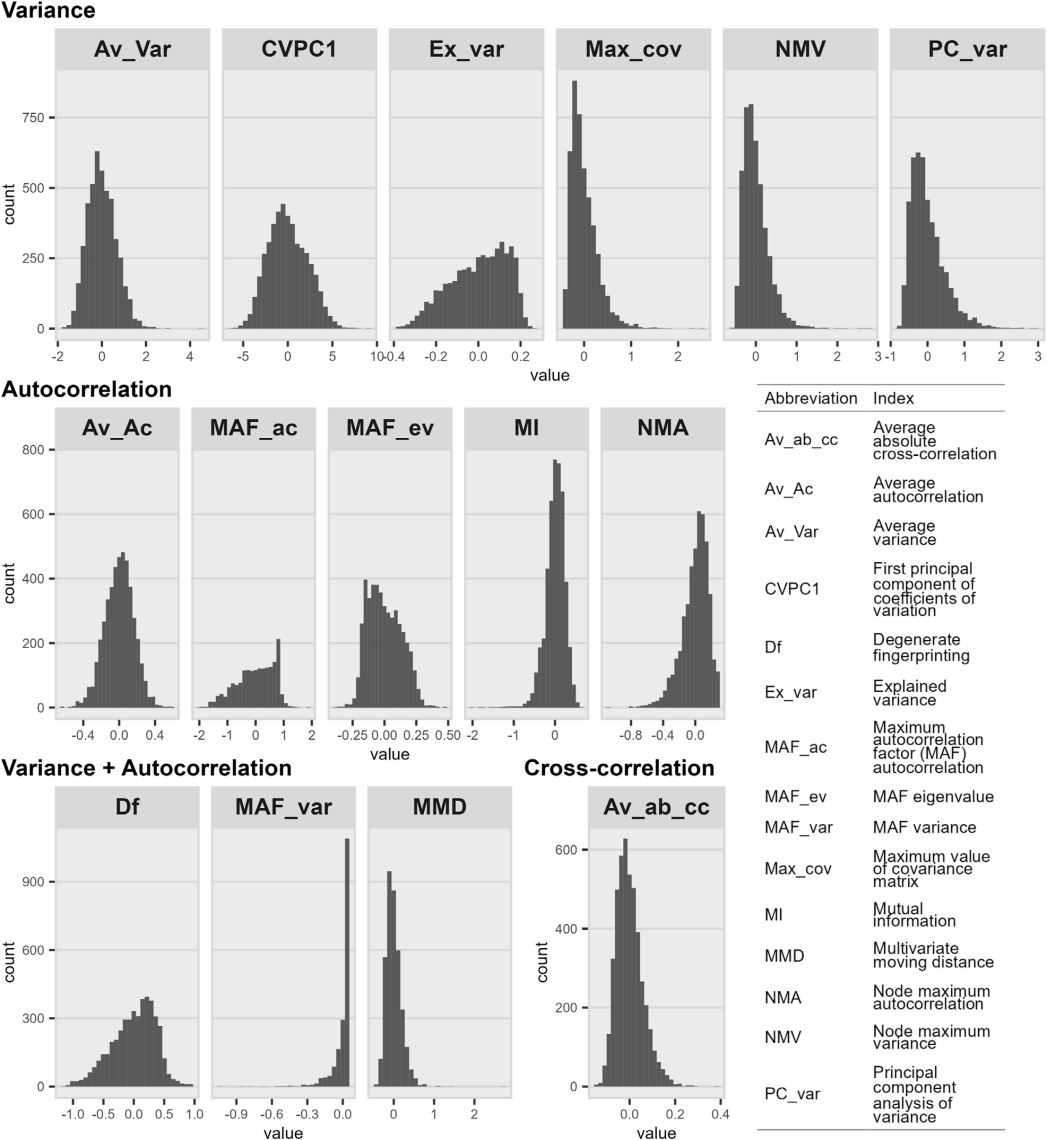
Histograms of index distributions organized by parameters on which they are based. Distributions are presented after indices were transformed and corrected for the number of observations included in the calculation. The number of calculated values was 4,550 for all indices except MAF_ac (*n* = 1,811), MAF_var (*n* = 1,811), and MMD (*n* = 3,756). As shown in Supplementary Figure S2, distributions further away from a normal one might impact the predictive power of the index.

Here, we aimed to compare these new indicators to other well-known EWSs, using the same list of indicators already compared in simulated data by Weinans et al. (2021; see Figure 1). We divided the EWSs based on the statistical parameters they are based on, namely variance, temporal AC (often referred to as lag-1 AC), cross-correlation, or a mix of the above. Then we assessed the correlations among the different indicators, their trends before death, and their performance in predicting mortality, using the same cohort of 763 CKD patients as before (Liu et al., 2021; Cohen et al., 2022).

We have demonstrated that MMD and CVPC1 are both effective predictors of all-cause mortality in this cohort (Liu et al., 2021; Cohen et al., 2022), all-cause mortality being used as a proxy of physiological collapse as a critical transition. Empirical datasets such as this one present advantages both on the practical and clinical sides. Data from patients on long-term hemodialysis represent easily accessible time series data, due to the regularity of blood measurements. Blood biomarkers, which can be used to construct EWSs, are measured roughly every 2 weeks as part of the routine clinical follow-up procedure. Moreover, in most cases, patients are kept on hemodialysis until kidney transplant or death, thus offering follow-up to the very end for a substantial subset. On the clinical relevance side, our previous work suggests that critical slowing down starts approximately 3 months prior to death in these patients (Cohen et al., 2022), allowing sufficient time for either corrective interventions or end-of-life planning.

## 2 Materials and methods

### 2.1 Data

The retrospective cohort used here has been described in detail elsewhere (Liu et al., 2021; Cohen et al., 2022). Briefly, it consists of electronic medical records extracted from the database of the CHUS hospital in Sherbrooke (Quebec, Canada) for all patients having had hemodialysis between 1997 and 2017. After excluding patients with acute kidney failure diagnosis, hemodialysis for less than 6 months, irregular visits and/or insufficient biomarker data, we retained 763 of the initial 2,565 patients. We restricted ourselves to the following 11 biomarkers with the highest time resolution, measured roughly every 2 weeks: hematocrit, hemoglobin, mean corpuscular hemoglobin (MCH), mean corpuscular hemoglobin concentration (MCHC), mean corpuscular volume (MCV), platelet count, potassium, red blood cell (RBC) count, red cell distribution width (RDW), sodium, and white blood (WBC) count.

### 2.2 Index calculation

We calculated 15 indices in total, 13 from Weinans et al. (2021; see Figure 1) and two from our previous work (Liu et al., 2021; Cohen et al., 2022; see Figure 1): one is based on cross-correlation, six on variance, five on AC, and three on both variance and AC. Indices were calculated as described before (Liu et al., 2021; Weinans et al., 2021; Cohen et al., 2022; see Supplementary Materials and Methods for details), every 6 months, starting from death or last available observation for censored individuals (or using a different time window if specified otherwise). Because MMD is calculated at each available observation rather than over a given time window, we averaged MMD values over the same time window (i.e., 6 months, unless specified otherwise) for ease of comparison with other indices. We also included all available MMD values (hereinafter referred to as “MMD_all”) in some cases to illustrate the effect of averaging on the index performance.

To calculate CVPC1, MMD, and NMV, biomarkers were first log- (glucose, RDW, and WBC) or square root- (platelet count) transformed to meet the assumption of an approximate normal distribution. We performed sensitivity analyses with and without biomarker transformations and for most indices, variable transformation did not significantly affect the results (see Supplementary Figure S1); however, for a few indices, there was an appreciable impact. In such cases, we selected index versions that seemed to best approach a normal distribution (for instance, see the effect of transforming variables beforehand on NMV calculation in Supplementary Figure S2). For all indices except CVPC1, biomarkers were then z-transformed to give similar importance to all biomarkers regardless of their scale. After their calculation, indices were also transformed themselves, if needed (see Supplementary Methods); nevertheless, some indices were still far from a normal distribution even after transformation (e.g., MAF_var; see Figure 1). Finally, we had shown before that CV values (in CVPC1 calculation) are biased by the number of observations included in the calculation (i.e., that CVs with fewer observations tended to be smaller), and had thus proposed a correction for this bias (Cohen et al., 2022). This problem emerges from irregularities in the data: although a blood test is prescribed every 2 weeks for patients on hemodialysis, the data we received from electronic medical records contained many gaps, leading to varying numbers of observations between individuals (and even between time intervals for a given individual). Here, we also tested if this bias was present for other indices too and, since it was the case, we controlled each index with the model that best fitted the values (see details in the Supplementary Materials and Methods).

### 2.3 Statistical analyses

We calculated Pearson correlations among each pair of indices. To visualize the trend before death, we plotted the average index values along with the 95% confidence interval (CI) in the 5 years preceding death for the subset of uncensored individuals (*n* = 511). We ran change point analyses on the indices calculated every 3 months and the “mcp” package (Lindeløv, 2020), allowing slopes to vary across individuals. Regression models were performed on all available index values, but results of the change point analyses are shown alongside trends before death calculated using 6-month time windows. We compared the performance of indices in predicting mortality with Cox proportional hazards models (coxph function from the survival package version 3.5–5; Therneau, 2020), controlling for age, sex, diabetes diagnostic, and length of follow-up. Age was modelled using a cubic spline with five degrees of freedom (“bs” function, “splines” package version 4.3.1). We clustered multiple observations per individual and included the square root of the number of observations used in each index value as a weight in the model, to account for the lower precision in estimation with fewer observations included in the calculation. Cox models were performed either using each index alone or combined all together into one model. We present hazard ratios as “HR95”, which represents the difference in hazard ratio (HR) between an individual at the 97.5th percentile and an individual at the 2.5th percentile of the index distribution. This approach was suggested to compare indices on different scales (Milot et al., 2014). Finally, we calculated the area under the receiver-operating characteristic (ROC) curve (AUC) for best performing indices, alone and in combination. To do so, we used the “roc” function from the “pROC” package, version 1.18.2 (Robin et al., 2011). We also assessed the effect of sequentially adding each index to control variables (the same as for the Cox models) on the AUC. The order in which we added the indices was determined through pairwise Pearson correlations (“correlate” and “rearrange” functions, “corrr” package version 0.4.4; Kuhn et al., 2022). All statistical analyses were performed with the R statistical language (Team, 2007) versions 4.1.3 (for index calculation and change point analyses) and 4.3.1 (for all other analyses) and source codes are available on the Cohen lab github page at https://github.com/cohenaginglab/EWS-comparison.

## 3 Results

### 3.1 Pairwise correlations among indices

**FIGURE 2:**
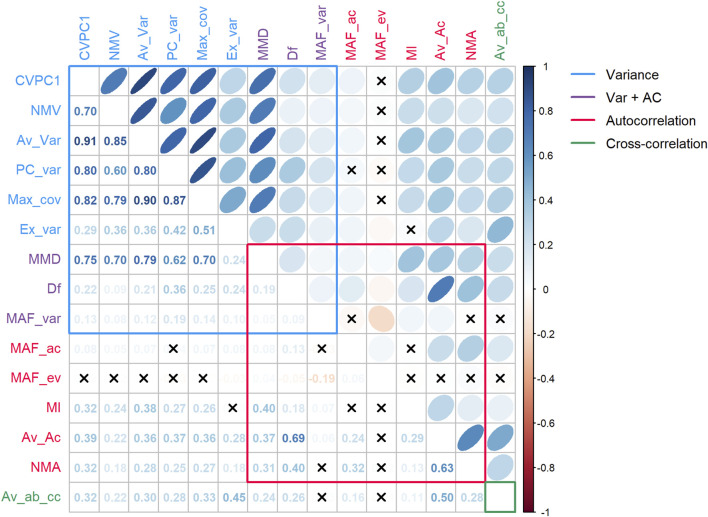
Pairwise correlations among indices. Indices were categorized according to the parameter(s) they are based on. Xs represent correlations not significant at α = 0.05. Abbreviations: AC, autocorrelation; Var, variance. Variance-based indices are mostly highly correlated with one another, whereas AC-based indices show poor to moderate correlations. Correlations among different index categories are at best moderate, suggesting that they capture, at least in part, different biological signals.

Figure 2 shows all pairwise correlations among indices, for which we obtained an overall mean *r* of 0.278 ± 0.255. Correlations were nearly always positive (96 out 105), and negative correlations were weak (all below −0.05, except for MAF_var vs. MAF_ev; *r* = −0.19, *p* < 0.001). Variance-based indices were more highly correlated with each other than indices based on AC (*r* = 0.663 and 0.170, respectively for indices based solely on variance or AC). The three indices based on both variance and AC all behave in different ways. MMD was highly correlated to most variance-based indices (mean *r* of 0.633, *p* < 0.001 for all six indices), particularly with Av_Var and CVPC1 (respectively *r* = 0.791 and 0.752), but to a lesser extent with Ex_var (*r* = 0.244). Df was highly correlated with Av_Ac (*r* = 0.693, *p* < 0.001) but much less strongly with all other indices (mean *r* of 0.235). On the other hand, MAF_var was poorly correlated with all other indices (mean *r* of 0.60). Finally, the only index based on cross-correlation (Av_ab_cc) was poorly to moderately correlated with other indices (mean *r* of 0.248), with the highest correlations found with Ex_var and Av_Ac (respectively *r* = 0.449 and *r* = 0.504, both with *p* < 0.001). When using shorter (2–4 months) or longer (1 year) time windows to calculate the indices, the pairwise correlations among them tended to get higher as the time window increased (Supplementary Figure S3), especially for a given set of indices (e.g., Ex_var, MMD, and NMA; see Supplementary Figure S4).

### 3.2 Trends before death and change point

**FIGURE 3:**
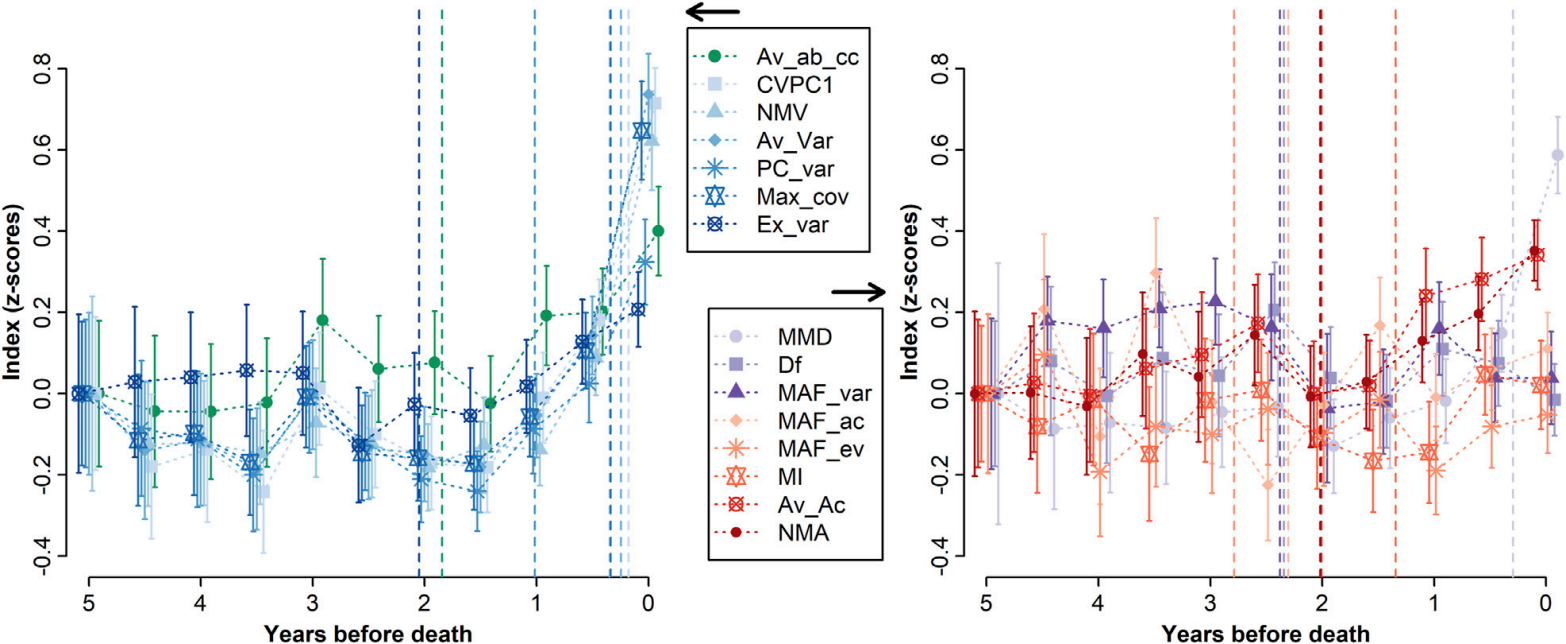
Trend before death for each index. Indices were averaged by time window, and means were plotted along with the 95% confidence intervals. All indices were z-transformed and centered at 5 years before death for ease of comparison, and were split into two graphs for ease of visualization. Vertical dashed lines indicate the results from change point analyses, i.e., the break point in values trend. Note that change point analyses were performed using the indices calculated with a 3-month time window for a better time resolution, although the results are plotted here against the 6-month trends. The best performing indices are all variance-based, except MMD, which is also based on AC.

Five indices showed a clear and sudden increase in the months preceding death (Figure 3): CVPC1, NMV, Av_Var, Max_cov, and MMD. The change point for these indices was roughly the same, at ∼3 months prior to death: 2.12 [0.00–7.11], 3.96 [2.70–5.58], 2.95 [0.00–9.64], 4.04 [0.00–10.92], and 3.55 [0.00–12.06] months, respectively for CVPC1, NMV, Av_Var, Max_cov, and MMD. Other indices showed either a moderate increase before death (e.g., PC_var) or no marked increase at all (e.g., MAF_ev). Trends were similar for indices calculated using shorter (2–4 months) or longer (1 year) time windows (Supplementary Figure S5). Nonetheless, for CVPC1, the increase was reduced for the version calculated with a 2-month time window compared with other versions. For NMV, Av_Var, and Max_cov, and MMD to a lesser extent, the opposite was true: the increase tended to be steeper with shorter time windows. MAF_ac and MAF_var could not be calculated with a 2-month time window since their computation required at least 13 observations per time window. Even with 3- and 4-month time windows, trends were very noisy due to the small number of observations per time point: 4 (IQR = 2, 6) and 9.5 (IQR = 4.75, 14.5), respectively.

### 3.3 Mortality prediction

**FIGURE 4:**
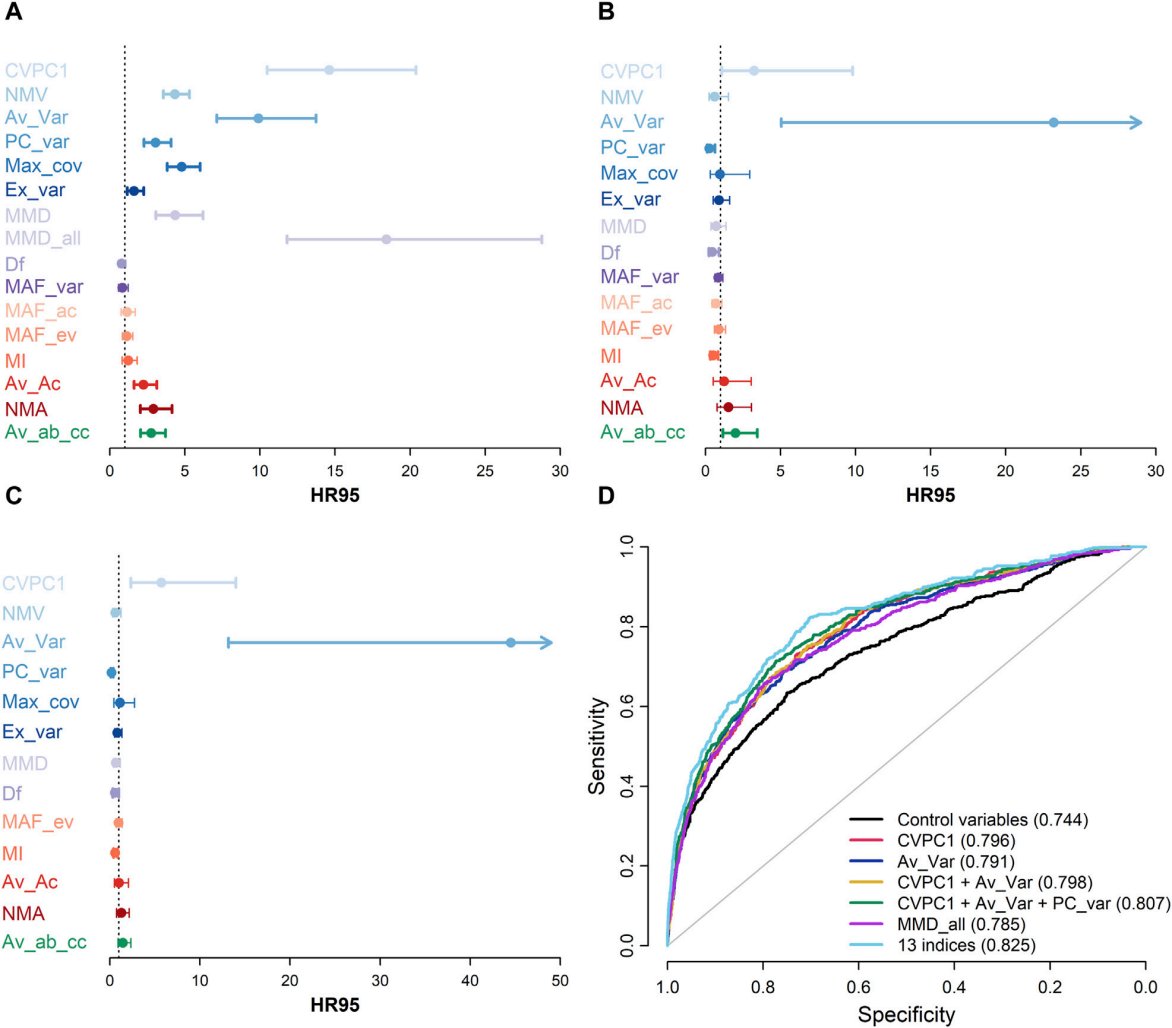
Mortality prediction for each index. A-C, HR95, i.e. the hazard ratio of being in the 97.5th percentile relative to the 2.5th percentile of the index, together with 95% confidence intervals are shown for each index in models including only this specific index (**(A)**, n ranges from 2,306 to 7,931 for all indices except MMD_all, for which *n* = 46,276), models including all indices (**(B)**, *n* = 2,270), and models including all indices except MAF_var and MAF_ac (**(C)**, *n* = 7,499). All models control for age using a cubic spline (with 5 degrees of freedom), sex, diabetes diagnosis, and length of follow-up, clustering multiple observations per individual. Hues of blue represent variance-based indices, hues of purple represent indices based on variance and auto-correlation, hues of red represent indices based on auto-correlation, and green represents the index based on cross-correlation. **(D)** Receiver operating characteristic (ROC) curves for a basic model including only demographic and control variables (black; age, sex, diabetes diagnosis, and length of follow-up) and for models including selected predictive indices (i.e., CVPC1, Av_Var, and PC_var), alone or in combination, are shown. Values for the area under the ROC curve are indicated in parentheses for each model in the legend. These results show that, despite a very high correlation, CVPC1 and Av_Var each contribute to predict mortality when included in the same model (Panels **(B,C)**), indicating subtle nuances in the signal captured by these indices because of computation differences. Also, although not overtly powerful when taken alone, other indices such as PC_var, seem to improve mortality prediction when added to multivariate models (Panels **(B–D)**).

Overall, results for mortality prediction were consistent with the observed trends before death (Figure 4). When included in separate Cox regression models (Figure 4A), the five indices with the steeper increase before death in Figure 3 also had the highest HR95 (i.e., the difference in HR between the 97.5th and the 2.5th percentile): 14.6 [10.5, 20.4], 9.9 [7.1, 13.7], 4.8 [3.8, 6.0], 4.4 [3.1, 6.2], 4.3 [3.6, 5.3], respectively for CVPC1, Av_Var, Max_cov, MMD, and NMV. In agreement with this, indices with moderate increase before death were also moderately powerful in predicting mortality; for example, PC_var had a HR95 = 3.0 [2.3, 4.1]. When unaveraged, MMD (“MMD_all”) was the strongest predictor of mortality, with a HR95 = 18.4 [11.8, 28.8]. However, when all indices were included in the same Cox model (Figure 4B), only Av_Var remained a strong predictor of mortality (HR95 = 23.2 [5.1, 106.5]), as well as CVPC1 and Av_ab_cc to a lesser extent (HR95 = 3.2 [1.1, 9.8] and 2.0 [1.2, 3.5], respectively). In the model controlling for all indices simultaneously, three indices became protective against mortality (reversed direction of effect): HR95 = 0.26 [0.11, 0.63], 0.46 [0.23, 0.91], and 0.52 [0.32, 0.84], respectively for PC_var, Df, and MI. This implies that high values of these indices contain two types of signals: a stronger signal, shared with the other indices, indicating high variability and high mortality risk, and a weaker signal unique to each of them indicating lower mortality risk. The analysis was repeated after excluding MAF_var and MAF_ac from the list (Figure 4C) since they both had a substantial proportion of missing values (n = 2,739, 60%). The results were consistent with the models including all 15 indices, with effects even stronger for Av_Var and CVPC1 (HR95 = 44.5 [13.2, 150.5] and 5.7 [2.3, 14.0], respectively), while Av_ab_cc no longer predicted mortality. PC_var and Df were still inversely associated with mortality risk (HR95 = 0.19 [0.09, 0.37] and 0.57 [0.33, 0.99], respectively), but not MI. Results were similar when using different time windows for index calculation (Supplementary Figure S6).

Figure 4D shows ROC curves and values for the AUC for models including only control variables (i.e., age, sex, diabetes diagnostic, and length of follow-up) or the best mortality predictors in Cox models (i.e., CVPC1, Av_Var, and MMD_all; Figure 4A), as well as a combination of: 1) CVPC1 and Av_Var; and 2) CVPC1, Av_Var, and PC_var. Combining CVPC1, Av_Var, and PC_var had the strongest effect on mortality prediction, raising the AUC to 0.81 compared with 0.74 for the basic model (i.e., including only control variables), and 0.79–0.80 with models including only one index. To understand better the effect of combining indices on mortality prediction, we calculated the change on the AUC when sequentially adding indices to the basic model (Supplementary Figure S7). Consistent with results from Cox models including all indices (see Figures 4B, C), Av_Var had the largest impact on the AUC (+0.035 in the model with 482 individuals and +0.059 in the model with 556 individuals), followed by PC_var (respectively +0.012 and +0.005 for both models) and CVPC1 (respectively +0.005 and 0.009 for both models). The analysis of Akaike information criteria also suggest some benefit to index combination: the AIC from the model with solely control variables is 32,127, compared to 24,722 for the model including all 13 indices (except MAF_var and MAF_ac) and to 31,117 for the model combining CVPC1, AV_Var, and PC_var (Supplementary Table S1).

## 4 Discussion

Here, we compared 15 multivariate indices proposed as EWSs of critical transitions (see Figure 1), using real-world data on biomarker dynamics preceding death in CKD patients on long-term hemodialysis. We grouped the indices based on their measurement of variance, AC, or cross-correlation. Broadly, we found that the variance-based indices correlated with each other and predicted mortality to a greater extent, whereas the other indices were at best weakly correlated with each other and weakly predicted mortality. Av_var is the single most promising index, performing well alone and even better in a full model containing all indices. Interestingly, CVPC1 and MMD – two ad-hoc variance indices (i.e., not derived based on mathematical theory) – also performed quite well, though the best version of MMD, MMD_all, could not be included in the full model due to the timescale at which it is calculated. This is, to our knowledge, the first comparison of different multivariate indices in real-world data, and demonstrates that even though in theory all the indices should perform well, in practice details of data structure (e.g., frequency or time-scale of measurement, missingness, sparseness, measurement error) do impact the performance of different indices.

While it would be tempting to conclude that variance-based indices are superior EWSs, that would be premature. The nature of the data used here was particularly sparse for time series data – measured at best every 2 weeks over several years – and this sparseness is more suited to variance-based indices (Dakos et al., 2012b). AC-based indices in this context may suffer from the fact that we do not know the optimal timescale to detect changes in AC in these biomarkers (seconds? minutes? days? weeks?) and further that the timescales may vary across biomarkers. However, others have found that the timescale did not matter when using AC to predict critical transitions in an ecological context (Batt et al., 2019). Collinearity among variables may also be considered when using AC-based EWSs (Cabrieto et al., 2018a), which has not been done here. Previous work in climate change even suggested that increase in AC might be harder to detect than increase in variance during critical slowing down (Ditlevsen and Johnsen, 2010). Only one cross-correlation index was available. The weak correlations among non-variance indices and their weak prediction of mortality may simply reflect that they have not been adequately measured. For instance, non-parametric approaches to measure cross-correlation appear to surpass parametric ones (Cabrieto et al., 2018b), while others cautioned against using cross-correlation indices in some contexts (Dean and Dunsmuir, 2016). This is also consistent with Weinans et al. (2021), which found that variance-based measures perform better than AC-based indices when using temporally sparse simulated data. On the other hand, variance-based measures may be more sensitive to stochastic effects near the threshold, as opposed to AC measures, which can lead to underestimation of the signal under an inadequate time window (Dakos et al., 2012b). In our study, CVPC1 appears to be the most sensitive index to the time window used (Supplementary Figures S5, S6), showing a decreased signal when calculated every 2 months compared to other time scales. Although we cannot rule out the impact of the fewer available observations for this shorter time window, it supports Dakos et al.’ work (2012b). Furthermore, in simple models where data resolution and data length are not limiting factors, we would expect Df and MAF_ac to be exactly the same, as well as MAF_var and PC_var. This happens because without these limitations we expect the first MAF and the first PC to be the same. The fact that in this study (Figure 2) they have a low correlation suggests that either the data are limited by length or resolution (especially affecting all indicators with an AC component) and/or that the noise is not equally distributed over variables.

Interestingly, the variance-based indices themselves were relatively well correlated but not completely redundant. Some were much stronger predictors of mortality than others. In a full model simultaneously controlling all indices for each other, Av_var was by far the strongest predictor, and indeed appears to summarize alone most of the key information contained in the ensemble, although CVPC1 still retained a substantial effect size in this model, implying at least some unique signal. This finding appears to somewhat contradict Ditlevsen and Johnsen’s work (2010), who proposed that signal from both variance and AC-based indices are needed to qualify as a true state transition rather than noise-induced shift. However, the finding that combining indices seems to improve mortality prediction is in line with previous work from Drake and Griffen (2010).

Somewhat puzzlingly, several indices changed the direction of their effect in the full model, such that they were protective against mortality rather than indicative of risk. This was never true for an index in a univariate model, so it implies that these indices – PC_var, MI, and Df – contain two types of signals, a stronger signal that is largely redundant with the ensemble and indicates risk of mortality, and a weaker, unique signal that indicates protection. The reason for this is unknown; we speculate that in physiological systems such as the one studied here, natural selection may have shaped dynamics that both mimic critical transitions and buffer against them via specific pathways. This would be reminiscent of what Chialvo (2008) proposed for the brain: the coexistence of mechanisms protective against critical transitions to avoid collapse, and mechanisms promoting them to facilitate adaptability. These indices might be detecting traces of such effects.

One question raised by our results is the extent to which critical slowing down, at least in physiological contexts such as the one studied here, is a single, unified phenomenon versus a series of interrelated but not fully unified processes. Work from non-biological contexts suggests various potential mechanisms leading to regime shifts (Dakos et al., 2015). In the former case, an appropriate dataset and measurement approach should provide at least the theoretical possibility of error-free measurement with a single index. In the latter, optimal prediction of critical transitions would inevitably require multiple indices used jointly. Put in other words, is there a “true” variability to the physiological system that is perfectly correlated with a “true” AC and a “true” cross-correlation, or are these phenomena somewhat distinct, and indeed perhaps more complex than these three simple categories? Our results weakly suggest the latter. Av_var is an exceptionally powerful predictor of mortality, but even with it in the model, CVPC1 still has a relatively strong effect. The inverse effects of several indices once overall variability is controlled for also suggest more nuanced dynamics. However, results are far from conclusive and further empirical and theoretical work is needed. Also, other approaches to physiological networks that focus on physiological function (Bashan et al., 2012), organ interactions (Bartsch et al., 2015), or hierarchical networks (Rizzo et al., 2020) might bring insightful perspective on this question. For instance, calculating EWSs within specific physiological systems (see Li et al., 2015) would offer valuable information on the overall system state and how they show signs of critical slowing down with respect to one another. Our previous findings showing striking synchrony across various physiological markers, including electrolytes, markers related to oxygen transport, and even immune functions (Cohen et al., 2022), tend to suggest that critical slowing down proceeds in a widespread manner across the various physiological organs and/or functions.

Theoretical considerations suggest clear relationships among the various indices proposed. However, real-world data are messy, containing missingness, sparseness, and various potential biases. It was not clear *a priori* whether the theoretical expectations would be observed in real-world data, and if not, to what extent the performance of different indices depends on particularities of the data set, or is generalizable. For example, it is possible that variance indices simply perform better across a range of contexts, and that Av_var is consistently a winner. Alternatively, this might depend on frequency of observations or timescales of variability in ways that will eventually become clear as other examples emerge. The worst-case scenario would be that the performance of different indices varies across contexts, but in ways that are hard to predict due to combinations of small biases in the data. This would mean it is not possible to come up with ways to choose some indices over other in novel contexts. In this sense, our study is a first attempt to understand how theoretical expectations play out in real-world data. Indeed, the poor performance of AC and cross-correlations here implies that the theoretical expectation of strong relationships among different types of indices is not upheld under our conditions of sparsity and missingness.

From a network physiology perspective, our results confirm the importance of integrative, multivariate approaches broadly, and more specifically suggest the importance of synchronicity. The best performing indices, Av_Var and CVPC1, can both be considered measures of the synchronized variability of biomarkers from distinct physiological compartments (i.e., with minimal correlations in the levels of the biomarkers). We think that these indices or other similar approaches could be useful for predicting and eventually understanding the dynamics of synchronization underlying physiological transitions and physiological collapse (Bartsch et al., 2015; Healy et al., 2021).

A combination of theory, simulations, and varied real-world datasets will be needed to understand optimal approaches to multivariate EWSs and the extent to which they do or do not depend on context. Nonetheless, our results provide broad support for the importance of variance-based metrics in predicting critical transitions, and point to some of the most promising ones. As additional examples accrue in real-world data, we expect a picture to emerge that can guide the choice of optimal indices for prediction: clear winners across contexts, context-dependence of winners, or unpredictability of winners.

## Data Availability

The data analyzed in this study is subject to the following licenses/restrictions: The dataset analyzed during the current study is not publicly available due to confidentiality concerns, transfer, and sharing of individual-level data, and thus requires prior approval from the CIRESSS and the Director of Professional Services of the CHUS, as well as by the Comité d’Éthique de la Recherche du CIUSSS de l’Estrie–CHUS. Requests to access these datasets should be directed to https://www.crchus.ca/en/services-outils/autres-services-et-outils/infocentre.
