# Supplementary material for: Application of early warning signs to physiological contexts: a comparison of multivariate indices in patients on long-term hemodialysis
Source: Front Netw Physiol. 2024 Mar 26;4:1299162. doi: 10.3389/fnetp.2024.1299162 (PMC11002238; doi:10.3389/fnetp.2024.1299162)
Supplement: Supplementary file 1 [file DataSheet1.pdf]

Supplementary Material

for

Application of early warning signs to physiological contexts: A comparison of multivariate indices in patients on long-term hemodialysis

Véronique Legault, Yi Pu, Els Weinans, and Alan A. Cohen

1 Supplementary Materials and Methods

1.1 Index calculation

For all indices, data were first divided into a given window for each individual, counting backwards from death or censoring, and then the calculations described below were performed. Whenever there was no variation in one biomarker within a time window due to laboratory rounding, we introduced a small random noise (with the “rnorm” function and a standard deviation of 0.001). For degenerate fingerprinting (Df), we first performed a principal component analysis (PCA) using the “prcomp” function on the whole dataset, and then calculated the autocorrelation (i.e., the Pearson correlation between values at one time point and the following one) of the first principal component (PC1) per individual and time window. The Minimum/maximum autocorrelation factor (MAF) autocorrelation (MAF_ac), eigenvalue (MAF_ec) and variance (MAF_var) were calculated using the “maf” function from the same named package (Haugen, M., 2022). For MAF_ac we extracted the autocorrelation for the first MAF factor, whereas for MAF_ec and MAF_var we calculated respectively the minimum and the variance of the first MAF factor. We used the “MutInf” function from the “DescTools” package (Signorell, A. et al., 2022) to calculate mutual information (MI) on values and their lagged version. Average autocorrelation (Av_ac) and node maximum autocorrelation (NMA) were respectively calculated as the mean and maximum correlation of all autocorrelations for individual variables. We computed node maximum variance (NMV) and average variance (Av_Var) respectively as the maximum and the mean value from all variances for individual variables. PCA variance (PC_var) was calculated as the variance from the PC1 performed on the whole cohort. The maximum value of the covariance matrix (Max_cov) was calculated as defined by its name, excluding the diagonal of the covariance matrix. Similarly, we excluded the diagonal from the correlation matrix for the computation of the average absolute cross-correlation (Av_ab_cc). To compute explained variance (Ex_var), we performed a PCA on the covariance matrix (with the “prcomp” function), and then calculated the mean of eigenvalues divided by their sum. For the PC1 of the coefficients of variation (CVs), we calculated the CVs for all variables by time window and individual, and then applied a PCA on all the CVs. Finally, to calculate the multivariate moving distance (MMD), we first calculated the covariance matrix with the complete cohort, and then used values from the preceding six months to calculate the vector of mean values in the Mahalanobis distance equation (Mahalanobis, 1936).

1.2 Index transformation and correction

After their computation, indices were transformed to better approach a normal distribution, except for Df, MAF_ev, MI, Av_Ac, NMA, Ex_var, and Av_ab_cc. CVPC1 and Av_Var were log-transformed, while we applied a square-root transformation to MMD, Max_cov, NMV, and PC_var. For MAF_ac and MAF_var, we used the following formula: $\log\,(\max(\mathrm{x}) + 0.01 - x)\ \times\ -1$.

After applying the transformations, we corrected the indices for the number of observations included in their calculation (as in Cohen et al., 2022), with the model that best fitted the data. For Av_ab_cc, we used $x = \frac{a}{n} + b$ (using the "nls" function), for MAF_ev and MAF_var, $x = \left(\frac{a}{\sqrt{n}}\right) + b$, for CVPC1, MMD, Av_Ac, Df, MI, Max_cov, and MAF_ac, $x = \left(\frac{1}{\sqrt{n}} \times a\right) + b$, and a linear model for Av_Var, Ex_var, NMA, NMV, and PC_var, where x is the index and n, the number of observations. Note that for CVPC1, however, the correction was applied on the CVs (for each biomarker), before performing the PCA.

2 Supplementary References

Cohen, A. A., Leung, D. L., Legault, V., Gravel, D., Blanchet, F. G., Côté, A.-M., et al. (2022). Synchrony of biomarker variability indicates a critical transition: Application to mortality prediction in hemodialysis. *iScience* 25, 104385. doi: 10.1016/j.isci.2022.104385.

Haugen, M. (2022). maf: Maximum Autocorrelation Factors. R package version 0.0.0.9000.

Mahalanobis, P. C. ; (1936). Mahalanobis distance. *Proc. Natl. Inst. Sci. India* 49, 234–256.

Signorell, A. et al. (2022). DescTools: Tools for descriptive statistics. R package version 0.99.46.

3 Supplementary Figures and Tables

3.1 Supplementary Figures

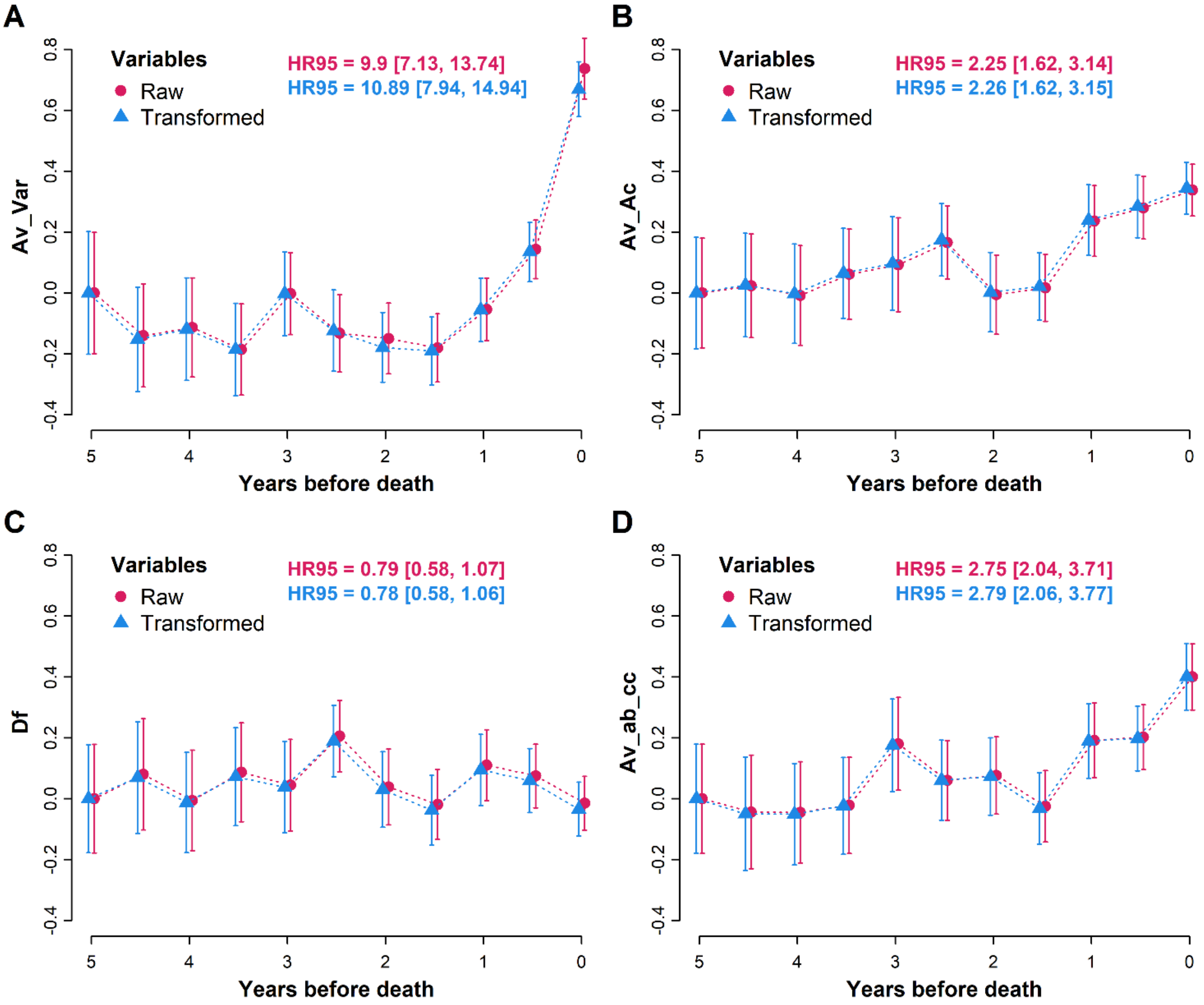

Supplementary Figure 1. Effect of transforming variables on four indices. Using either raw (pink) or transformed (blue) biomarkers had very little effect on the trend before death and mortality prediction for most indices. Here, the results are shown for Av_Var (**A**), Av_Ac (**B**), Df (**C**), and Av_ab_cc (**D**). All indices were z-transformed and centered at five years before death for ease of comparison, and means per 6-month time window are shown, along with the 95% confidence intervals.

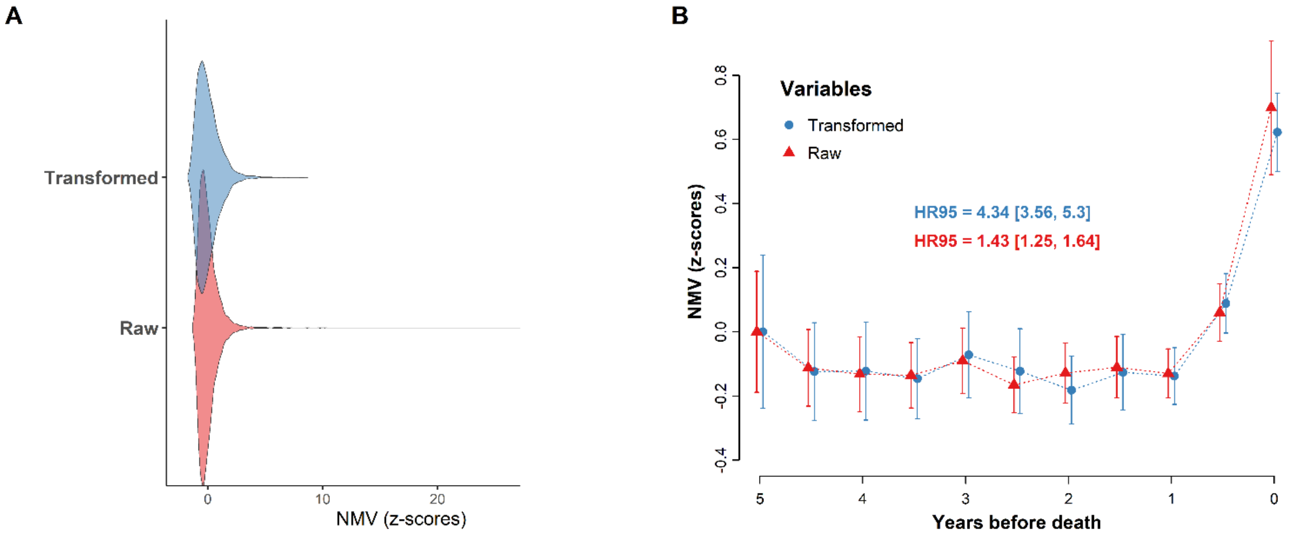

Supplementary Figure 2. Effect of transforming variables on NMV calculation. A, NMV distribution calculated using raw variables (in red) is substantially more right-skewed, driven by a few extreme values (the three NMV values above 4 were all measured within six months from death), compared to the version calculated with transformed variables (in blue), so that variables are approximately normally distributed before NMV computation. **B**, Both NMV versions were z-transformed and centered at five years before death for ease of comparison, and means per 6-month time window are shown, along with the 95% confidence intervals. Although trends before death are similar for both versions, NMV calculated using transformed variables (in blue) is more powerful in predicting mortality (HR95 = 4.34), compared to the version calculated with raw variables (in red, HR95 = 1.43).

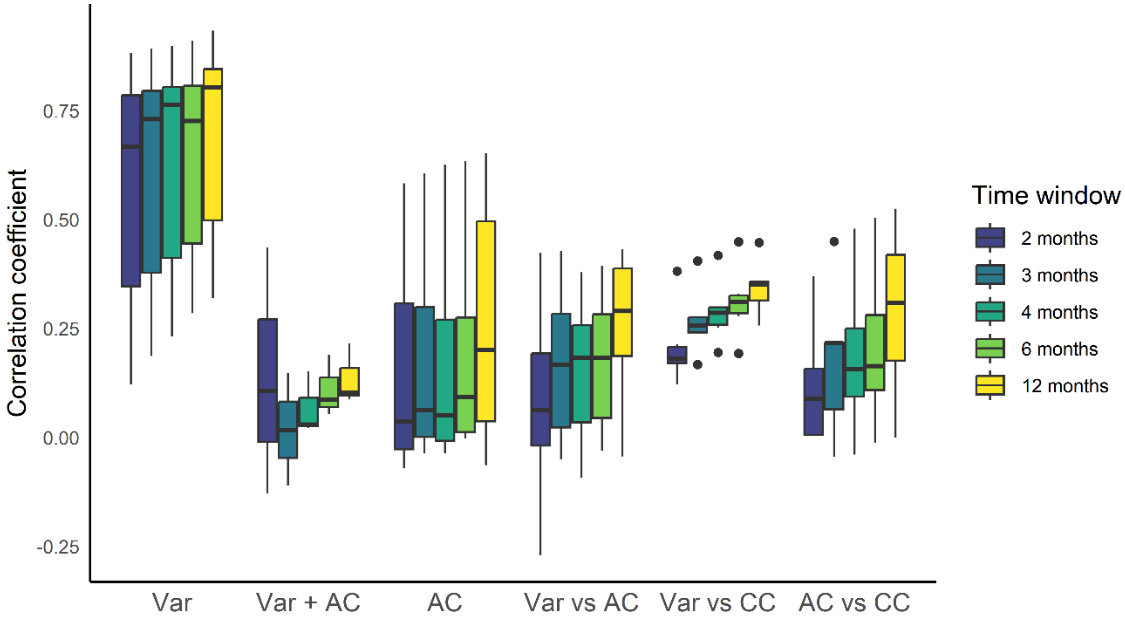

Supplementary Figure 3. Change in pairwise correlations among indices according to the time windows used in the calculation. Pearson correlations were calculated between indices computed using time windows of 2, 3, 4, and 12 months. Within each box, the hinges represent the 25th and 75th percentiles of correlation coefficients, while the horizontal line represents the median. The whisker length represents 1.5 times the interquartile range and outliers are shown by individual dots. Individual correlations are shown in Fig. S4. Abbreviations: AC, autocorrelation; CC, cross-correlation; Var, variance.

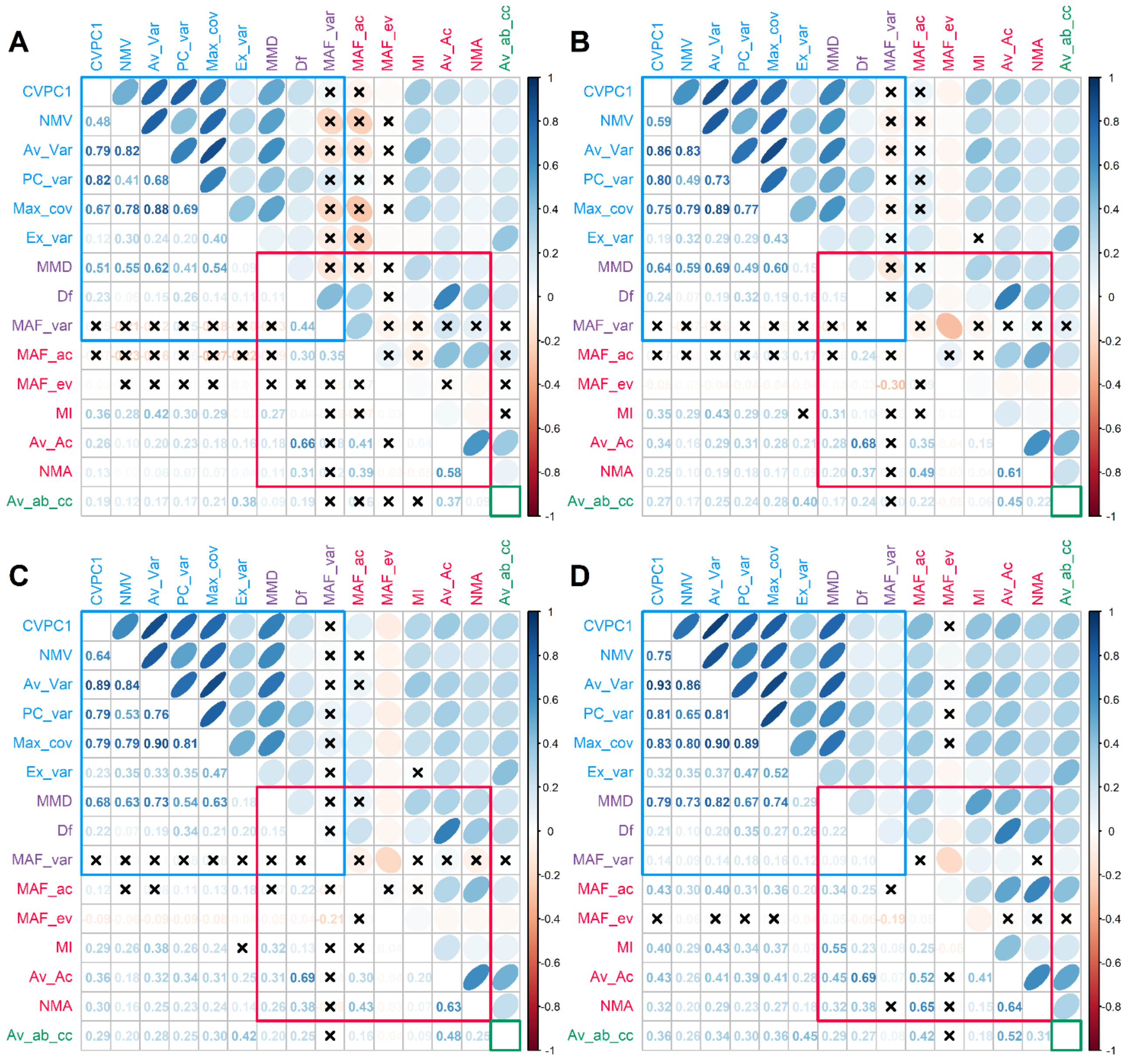

Supplementary Figure 4. Pairwise correlations among indices calculated using different time windows. Indices were calculated using a 2-month (**A**), 3-month (**B**), 4-month (**C**), or 1-year (**D**) time window. Within each panel, indices are categorized according to the parameter(s) they are based on (i.e., blue for variance-based indices, purple for indices based on both variance and autocorrelation, red for autocorrelation-based indices, and green for the index based on cross-correlation). Xs represent correlations not significant at $\alpha = 0.05$.

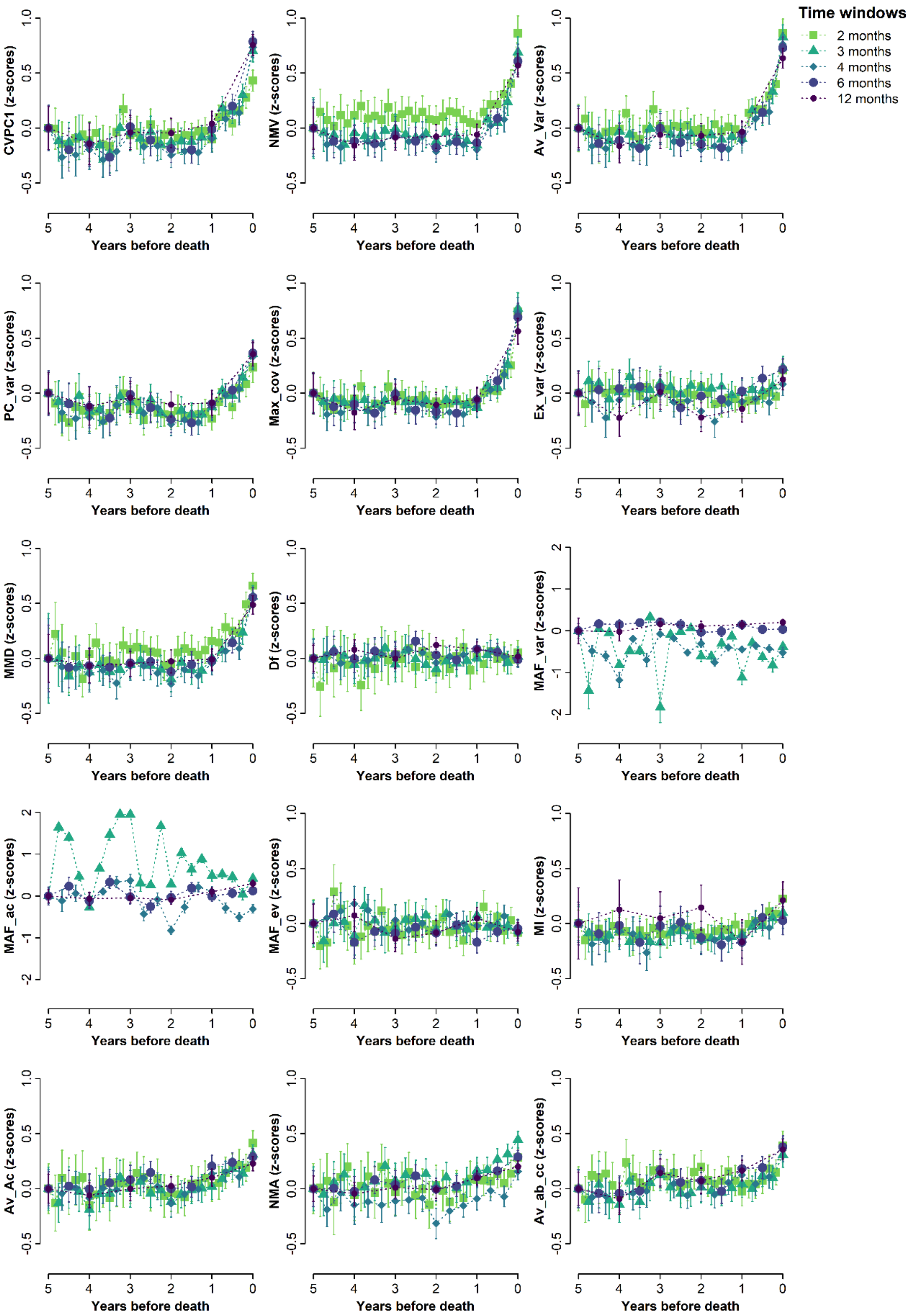

Supplementary Figure 5. Trend before death for each index, by time window used in the calculation. All indices were z-transformed and centered at five years before death for ease of comparison and to facilitate change over time visualization. Means per time window are shown, along with the 95% confidence intervals. All y-axes are the same, except for MAF_var and MAF_ac.

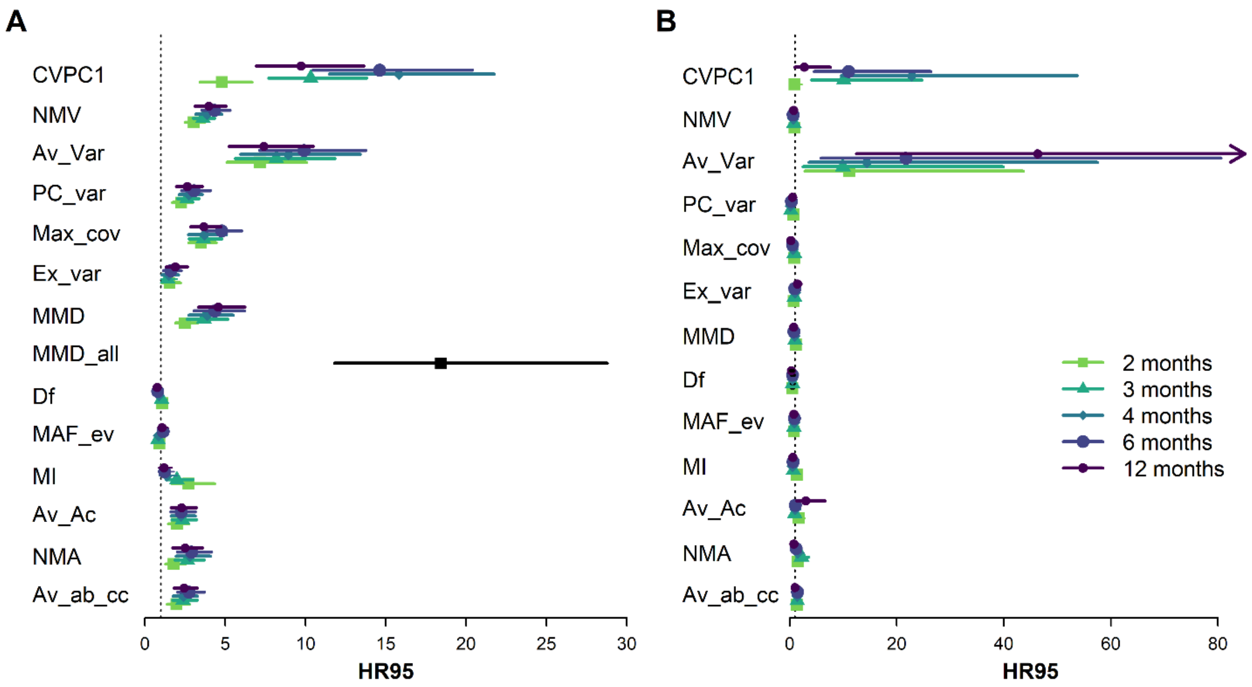

Supplementary Figure 6. Mortality prediction for each index by time window used in the calculation. HR95, i.e. the hazard ratio of being in the 97.5th percentile relative to the 2.5th percentile of the index, together with 95% confidence intervals are shown for each index in models including only this specific index (**A**) and models including all indices except MAF_var and MAF_ac (**B**). All models control for age using a cubic spline (with 5 degrees of freedom), sex, diabetes diagnosis, and length of follow-up, clustering multiple observations per individual. Arrows represent confidence intervals larger than the x-axis limits.

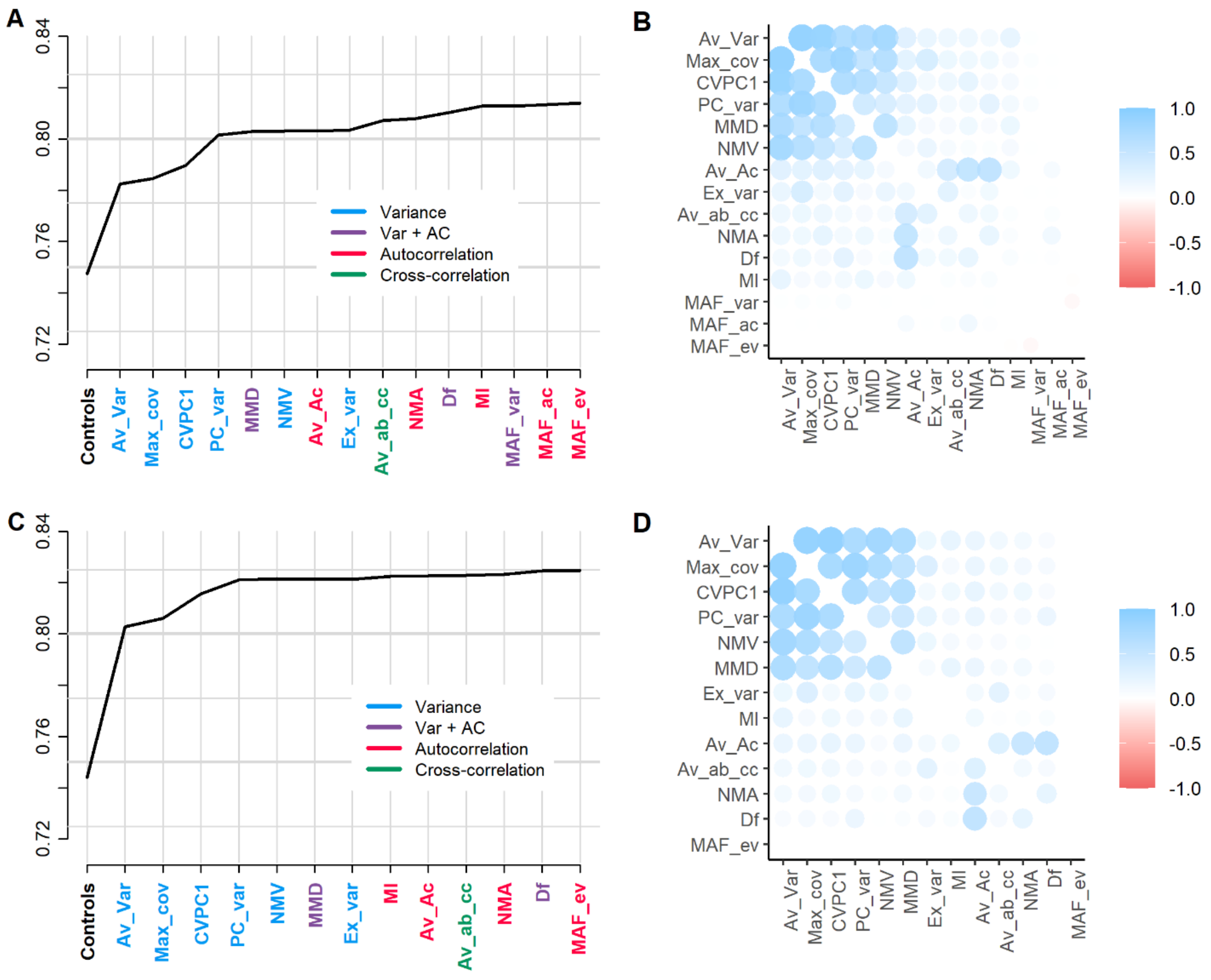

Supplementary Figure 7. Effect of combining indices on the area under the receiving operator characteristic curve (AUC). **A-C**; All indices were sequentially added to Av_Var and the change on the AUC is shown on the y-axis, with (**A**, n = 482 individuals) or without including MAF_ac and MAF_var (**C**, n = 556 individuals). Controls include age (modelled using a cubic spline with five degrees of freedom), sex, diabetes diagnosis, and length of follow-up. Different colors indicate different categories of indices. **B-D**; Indices were ordered based on their pairwise correlations.

3.2 Supplementary Table

Supplementary Table 1. Akaike information criterion (AIC) values for all Cox models predicting mortality.

Indices included in model	AIC
**None (control variables only, n = 3,756)**	**32,127**
CVPC1	31,317
NMV	31,686
Av_Var	31,408
PC_var	31,952
Max_cov	31,647
Ex_var	32,098
MMD	25,446
Df	32,122
MAF_ev	32,126
MI	32,123
Av_Ac	32,056
NMA	32,024
Av_ab_cc	31,990
CVPC1+ Av_Var	31,294
CVPC1+ Av_Var + PC_var	31,117
All indices, except MAF_var and MAF_ac	24,722
**None (control variables only, n = 1,653)**	**17,871**
MAF_var	19,462
MAF_ac	19,465
All 15 indices	17,348
**None (control variables only, n = 46,276)**	**8,473**
MMD_all	8,178
